# Risk Factors for Mortality in Anti-NMDAR, Anti-LGI1, and Anti-GABABR Encephalitis

**DOI:** 10.3389/fimmu.2022.845365

**Published:** 2022-03-07

**Authors:** Rui Zhong, Qingling Chen, Xinyue Zhang, Hanyu Zhang, Weihong Lin

**Affiliations:** ^1^ Department of Neurology, The First Hospital of Jilin University, Changchun, China; ^2^ Department of Hepatology, Second People’s Clinical College of Tianjin Medical University, Tianjin, China

**Keywords:** mortality, autoimmune encephalitis, anti-NMDAR encephalitis, anti-LGI1 encephalitis, anti-GABABR encephalitis

## Abstract

**Objective:**

We aimed to investigate the mortality rate and identify the predictors of death in patients with anti-NMDAR, anti-LGI1, and anti-GABABR encephalitis.

**Methods:**

Patients with anti-NMDAR, anti-LGI1, and anti-GABABR encephalitis were recruited from the Neurology Department of the First Hospital of Jilin University from March 2015 to November 2021. The primary outcome variable was a binary variable of death vs. survival. The potential risk factors for mortality were evaluated. The mortality rates were determined, and the independent predictors of death were identified using multivariable logistic regression analysis.

**Results:**

A total of 100 hospitalized patients with anti-NMDAR, anti-LGI1, or anti-GABABR encephalitis were included in the final analysis. Fifteen patients (15%) died during a median follow-up period of 18 months. The mortality rates were 10% for anti-NMDAR encephalitis, 2.8% for anti-LGI1 encephalitis, and 41.7% for anti-GABABR encephalitis. The multivariable analysis results showed that older age at onset [adjusted odds ratio (OR) = 1.017, 95% confidence interval (CI) = 1.009–1.136; *p* = 0.023] was independently associated with an increased risk of death. Antibody type was also associated with mortality. Patients with anti-GABABR encephalitis had 13.458-fold greater odds of dying than patients with anti-LGI1 encephalitis (adjusted OR = 13.458, 95% CI = 1.270–142.631; *p* = 0.031).

**Conclusion:**

The general mortality rate of anti-NMDAR, anti-LGI1, and anti-GABABR encephalitis was 15%. Age at onset and type of autoimmune encephalitis antibody were independent predictors of death in these patients.

## Introduction

In recent years, the group of neuroinflammatory diseases known as autoimmune encephalitis (AE) has been recognized to encompass an increasingly wide and diverse range of pathological processes associated with the presence of antibodies against neuronal intracellular proteins, synaptic receptors, ion channels, and/or neuronal surface proteins (1–3). Many types of AE have been discovered in recent decades due to the development of biochemical assays; the most common types of neuronal surface antibody (NSAb)-associated AE include anti-*N*-methyl-D-aspartate receptor (NMDAR), anti-γ-aminobutyric acid B receptor (GABABR), and anti-leucine-rich glioma-inactivated protein 1 (LGI1) encephalitis (3, 4). Immunotherapy improves the prognosis of most AE patients (5, 6) and is related to a decreased risk of clinical relapses of AE (7–9).

Mortality in AE patients has received little attention (10, 11). Hong and her colleagues reported high mortality in patients with anti-GABABR encephalitis; 32.1% of these patients died during the disease course (10). The mortality rate of anti-NMDAR encephalitis as reported by prior studies ranged from 5% to 11.46%, which was lower than that of anti-GABABR encephalitis (7, 11, 12). The predictors of death in AE patients also varied across prior studies (10, 11). The following clinical variables were identified as risk factors for death: number of complications, intensive care unit (ICU) admission, length of ICU stay, age at onset, presence of a tumor, and deep venous thrombosis (10, 11, 13).

Data on the analysis of mortality in patients with anti-NMDAR, anti-LGI1, and anti-GABABR encephalitis are lacking in Northeast China. Thus, we aimed to delineate the mortality rate in these patients and identify potential factors associated with death.

## Methods

### Study Design and Participants

This retrospective observational study was conducted at a tertiary hospital serving a population of more than 5,000,000 per year in Northeast China. Hospitalized patients with anti-NMDAR, anti-LGI1, and anti-GABABR encephalitis were recruited from the Neurology Department of the First Hospital of Jilin University from March 2015 to November 2021. The diagnosis of AE was confirmed in accordance with the diagnostic criteria established in 2016 (14). Cerebrospinal fluid (CSF) samples from all suspected AE patients were tested for AE antibodies using cell-based assays. These assays were performed with CSF rather than serum due to the superior quality of CSF samples. The other inclusion criteria were as follows: 1) the AE case must be newly diagnosed and 2) the patient must be in the acute phase of AE (defined as the first 3 months after the onset of AE symptoms). The exclusion criteria were as follows: 1) clinical and laboratory evidence of infectious encephalitis, caused by an agent such as *Mycobacterium tuberculosis* (TB) or a virus; 2) presence of other types of AE antibodies or neurological paraneoplastic antibodies; and 3) a follow-up period shorter than 6 months. The Ethics Committee of our hospital granted ethical approval for the retrospective observational study and waived the requirement for written informed consent. This study was performed in accordance with the Declaration of Helsinki.

### Data Collection

For each eligible patient, the following demographic and clinical data were gathered retrospectively from our hospital electronic medical records and nursing records: gender, age at onset, clinical symptoms, presence of tumors, 24-h video-electroencephalography (video-EEG) reporting, brain MRI findings, CSF analysis, antibody type, antibody titer, immunotherapy, and hospitalization data.

### Definition

According to the International League Against Epilepsy (ILAE), tonic–clonic status epilepticus (SE) is defined as bilateral tonic–clonic activity lasting longer than 5 min, and absence SE and focal SE are defined by the occurrence of their respective clinical signs for more than 10 min (15). We adopted these definitions in the current study. Immunotherapy was considered to be delayed if the regimen was started at least 28 days after AE symptom onset (16, 17). We defined a long hospital stay as a hospital length of stay >30 days. Clinical relapse of AE was defined as new onset or worsening of symptoms occurring after an initial improvement or stabilization lasting at least 2 months (14). A CSF antibody titer ≤1:10 was scored as (+), values >1:10 but ≤1:100 as (++), and values >1:100 as (+++). Hyperintensity on T2-weighted imaging (T2WI) and fluid-attenuated inversion recovery (FLAIR) imaging and hypointensity on T1-weighted imaging (T1WI) were defined as abnormal brain MRI. Abnormal EEG was defined as the occurrence of interictal epileptic discharges such as spikes or sharp waves. CSF analysis results were classified as abnormal or normal based on reference intervals.

### Outcome Assessment

The primary outcome variable was a binary variable of death vs. survival. AE patients were followed up by clinic visits or telephone interviews for at least 6 months after discharge. The primary outcome assessments were performed at 1, 3, and 6 months after discharge and then every 6 months thereafter. Follow-up ended when the patient died during the disease course or was lost to follow-up.

### Statistical Analysis

Continuous variables are presented as medians [interquartile ranges (IQRs)], and categorical variables are expressed as numbers and percentages. Continuous variables were compared using the Mann–Whitney *U* tests between two groups or the Kruskal–Wallis tests between three groups, and Pearson’s chi-squared test or Fisher’s exact test was used for categorical variables. Variables with *p <*0.05 in univariate analysis were retained in multivariable analysis. A multivariable logistic regression analysis was employed to identify the independent predictors of death, and the results are described as odds ratios (ORs) with 95% confidence intervals (CIs). Subgroup analyses based on AE type were performed if there were more than three patients who died. All reported *p*-values were two-tailed and considered statistically significant at <0.05. All data were analyzed using IBM SPSS version 26.0 (SPSS, Chicago, IL, USA).

## Results

### Patient Characteristics

**Figure 1:**
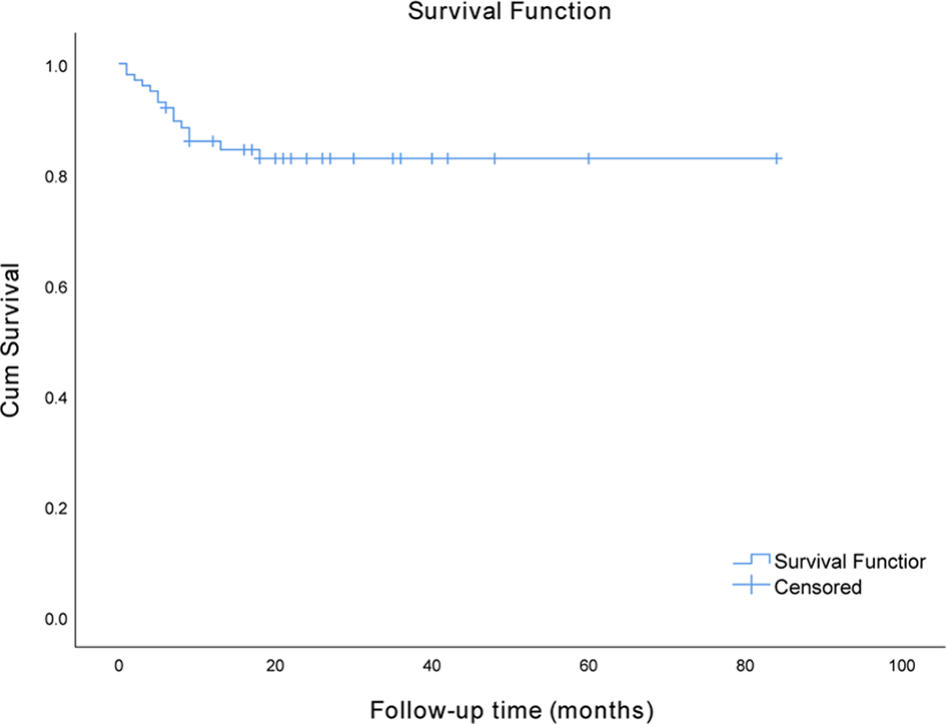
Analysis of the Kaplan–Meier survival curve for survival.

In total, 100 hospitalized patients with anti-NMDAR, anti-LGI1, and anti-GABABR encephalitis were included in the final analysis after 10 patients were excluded based on the exclusion criteria. Among these patients, 40 had anti-NMDAR encephalitis, 36 had anti-LGI1 encephalitis, and 24 had anti-GABABR encephalitis. The demographic and clinical characteristics of these patients are described in **Table 1**. In this cohort, which consisted of 54 men (54.0%) and 46 women (46.0%), the median age at onset was 52 years. Only one patient did not receive immunotherapy; however, immunotherapy was delayed in half of the patients. Forty-five (45%) patients required admission to the ICU in the hospital. Eventually, 15 (15%) patients died in the course of the disease. The median time interval from onset to death in the patients who died was 6 months, and the range was 1 to 18 months. The survival curve of the study participants is presented in **Figure 1**.

**Table 1:** Patient characteristics by type of AE.

Characteristics	Total (*n* = 100)	NMDAR (*n* = 40)	LGI1 (*n* = 36)	GABABR (*n* = 24)	*p*-value
Men	54 (54.0)	22 (55.0)	19 (52.8)	13 (54.2)	0.981
Age at onset (years) (median, IQR)	52 (33, 63)	31 (21, 42)	58 (49, 66)	62 (56, 65)	<0.001
Tumor presence	11 (11.0)	5 (12.5)	1 (2.8)	5 (20.8)	0.084
EEG (abnormal)	57 (57.0)	19 (47.5)	22 (61.1)	16 (66.7)	0.268
Brain MRI (abnormal)	55 (55.0)	20 (50.0)	25 (69.4)	10 (41.7)	0.076
CSF analysis (abnormal)	86 (86.0)	36 (90.0)	28 (77.8)	22 (91.7)	0.203
Antibody titer					
+	31 (31.0)	17 (42.5)	10 (27.8)	4 (16.7)	0.26
++	51 (51.0)	17 (42.5)	20 (55.6)	14 (58.3)	
+++	18 (18.0)	6 (15.0)	6 (16.7)	6 (25.0)	
Administration of HGG	85 (85.0)	37 (92.5)	24 (66.7)	15 (62.5)	0.006
Administration of corticosteroids	76 (76.0)	35 (87.5)	31 (86.1)	19 (79.2)	0.647
Administration of plasma exchange	5 (5.0)	4 (10.0)	0	1 (4.2)	0.133
Second-line immunotherapy	6 (6.0)	4 (10.0)	2 (5.6)	0	0.262
Without immunotherapy	1 (1.0)	0	0	1 (4.2)	0.202
Immunotherapy delay	50 (50.0)	14 (35.0)	27 (75.0)	9 (37.5)	0.001
ICU admission	45 (45.0)	24 (60.0)	5 (13.9)	16 (66.7)	<0.001
Relapse	26 (26.0)	10 (25.0)	8 (22.2)	8 (33.3)	0.619
Death	15 (15.0)	4 (10.0)	1 (2.8)	10 (41.7)	<0.001
Follow-up time (median, IQR)	18 (10, 30)	15 (6, 24)	24 (17, 36)	12 (6, 26)	0.001

AE, autoimmune encephalitis; IQR, interquartile range; NMDAR, N-methyl-D-aspartate receptor; LGI1, leucin-rich glioma inactivated-1; GABABR, g-aminobutyric acid type B receptor; CSF, cerebrospinal fluid; EEG, electroencephalogram; MRI, magnetic resonance imaging; HGG, human gamma globulin; ICU, intensive care unit. +: antibody titer ≤1:10; ++: antibody titer >1:10 and ≤1:100; +++: antibody titer >1:100 as (+++).

### Characteristics of Different Types of AE

**Table 1** also shows the patient characteristics by antibody type. Patients with anti-NMDAR encephalitis had the youngest age at onset (31 vs. 58 vs. 62 years, *p* < 0.001). Patients with anti-GABABR encephalitis had the highest prevalence of tumors; however, this difference was not statistically significant (12.5% vs. 2.8% vs. 20.8%, *p* = 0.084). Patients with anti-LGI1 encephalitis were less likely to require admission to the ICU than those with anti-NMDAR or anti-GABABR encephalitis (60.0% vs. 13.9% vs. 66.7%, respectively; *p* < 0.001). The case fatality rate also differed significantly among these three groups (10.0% vs. 2.8% vs. 41.7%, respectively; *p* < 0.001).

### Univariate Analysis of Predictors of Death

The median follow-up time was 18 months (IQR 10–30, range 6–84 months). During the follow-up period, 15 AE patients (15%) died. Comparisons of variables between the death group and the survival group are shown in **Table 2**. The age at onset in the death group was 63 years, which was significantly older than the onset age of 48 years in the survival group (*p* = 0.009). The occurrence of SE was more common in the death group than in the survival group (66.7% vs. 37.6%, *p* = 0.036). Patients with anti-GABABR encephalitis had an increased risk of death (66.7% for anti-GABABR vs. 16.5% overall for the two other types; *p* < 0.001). ICU admission was significantly associated with an increased risk of mortality (73.3% vs. 40.0%, *p* = 0.017). No other variable was found to be associated with mortality in this cohort of patients with anti-NMDAR, anti-LGI1, and anti-GABABR encephalitis.

**Table 2:** Univariate analysis of variables associated with death in the overall sample of patients.

Variables	Death (*n* = 15)	Survival (*n* = 85)	*p*-value
Men	11 (73.3)	43 (50.6)	0.103
Age at onset (years) (median, IQR)	63 (52, 68)	48 (31, 60)	0.009
Acute symptomatic seizure	13 (86.7)	74 (87.1)	1
Status epilepticus	10 (66.7)	32 (37.6)	0.036
Fever (>37.5°C)	6 (40.0)	28 (42.9)	0.595
Psychiatric symptoms and behavior disorders	10 (66.7)	54 (63.5)	0.815
Movement disorders	3 (20.0)	34 (40.0)	0.139
Sleep disorders	8 (53.3)	31 (36.5)	0.217
Autonomic disturbance	5 (33.3)	39 (45.9)	0.367
Tumor presence	4 (26.7)	7 (8.2)	0.058
EEG (abnormal)	9 (60.0)	48 (56.5)	0.799
Brain MRI (abnormal)	8 (53.3)	47 (55.3)	0.888
CSF analysis (abnormal)	13 (86.7)	73 (85.9)	1
CSF cell count (abnormal)	8 (53.3)	43 (50.6)	1
CSF protein level (abnormal)	4 (26.7)	26 (30.6)	1
Antibodies			
NMDAR	4 (26.7)	36 (42.4)	<0.001
LGI1	1 (6.7)	35 (41.2)	
GABABR	10 (66.7)	14 (16.5)	
Antibody titer			
+	2 (13.3)	29 (34.1)	0.264
++	10 (66.7)	41 (48.2)	
+++	3 (20.0)	15 (17.6)	
Administration of HGG	9 (60.0)	67 (78.8)	0.213
Administration of corticosteroids	10 (66.7)	75 (88.2)	0.078
Administration of plasma exchange	0	5 (5.9)	1
Second-line immunotherapy	0	6 (7.1)	0.587
Without immunotherapy	1 (6.7)	0	0.15
Immunotherapy delay	6 (40.0)	44 (51.8)	0.401
Internal to immunotherapy	25 (13, 40)	30 (14, 43)	0.653
ICU admission	11 (73.3)	34 (40.0)	0.017
Mechanical ventilation	4 (26.7)	14 (16.5)	0.464
Long hospital stay	4 (26.7)	27 (31.8)	0.772
Relapse	2 (13.3)	24 (28.2)	0.371

IQR, interquartile range; NMDAR, N-methyl-D-aspartate receptor; LGI1, leucin-rich glioma inactivated-1; GABABR, g-aminobutyric acid type B receptor; CSF, cerebrospinal fluid; EEG, electroencephalogram; MRI, magnetic resonance imaging; HGG, human gamma globulin; ICU, intensive care unit. +: antibody titer ≤1:10; ++: antibody titer >1:10 and ≤1:100; +++: antibody titer >1:100 as (+++).

### Multivariable Analysis of Predictors of Death

A univariate analysis was performed to identify the variables associated with death in AE patients. Age at onset, occurrence of SE, antibody type, and ICU admission met the significance threshold of *p <*0.05 and were retained in the multivariable stepwise logistic regression models. The multivariable analysis results showed that older age at onset (adjusted OR = 1.017, 95% CI = 1.009–1.136; *p* = 0.023) was independently associated with an increased risk of death. Antibody type was also associated with death. The odds of dying were 13.458 times greater in patients with anti-GABABR encephalitis than in patients with anti-LGI1 encephalitis (adjusted OR = 13.458, 95% CI = 1.270–142.631; *p* = 0.031). The results are described in **Table 3**. No independent association was found between a history of SE or ICU admission and death (*p* > 0.05).

**Table 3:** Multivariable analysis of variables independently associated with death in the overall sample of patients.

Variables	Adjusted OR	95% CI	*p*-value
Age at onset	1.071	1.009–1.136	0.023
Status epilepticus	1.928	0.445–8.355	0.38
Antibodies type			0.093
Antibodies type (1)	11.974	0.806–177.892	0.071
Antibodies type (2)	13.458	1.270–142. 631	0.031
ICU admission	2.281	0.478–10.876	0.301

ICU, intensive care unit; OR, odds ratio; CI, confidence interval.

### Subgroup Analysis

A subgroup analysis was performed based on AE antibody types. The risk factors for mortality in the subgroup with anti-LGI1 encephalitis were not investigated because only one patient died during the disease course. In anti-NMDAR encephalitis, no factor was found to be associated with the risk of death. As for patients with anti-GABABR encephalitis, a significant association was observed between older age at onset and a higher risk of mortality (*p* = 0.016). For details, see **Table 4**.

**Table 4:** Univariate analysis of variables associated with death in patients with anti-NMDAR and anti-GABABR encephalitis.

Variables	NMDAR (*n* = 40)		*p*-value	GABABR (*n* = 24)		*p*-value
	Death (*n* = 4)	Survival (*n* = 36)		Death (*n* = 10)	Survival (*n* = 14)	
Men	3 (75.0)	19 (52.8)	0.751	7 (70.0)	6 (42.9)	0.368
Age at onset (years) (median, IQR)	45 (24, 67)	30 (20, 40)	0.176	64 (62, 68)	57 (53, 61)	0.016
Acute symptomatic seizure	3 (75.0)	30 (83.3)	0.552	9 (90.0)	13 (92.9)	1
Status epilepticus	2 (50.0)	13 (36.1)	1	7 (70.0)	9 (64.3)	1
Fever (>37.5°C)	3 (75.0)	18 (50.0)	0.673	2 (20.0)	6 (42.9)	0.464
Psychiatric symptoms and behavior disorders	3 (75.0)	26 (72.2)	1	7 (70.0)	9 (64.3)	1
Movement disorders	1 (25.0)	22 (61.1)	0.249	2 (20.0)	2 (14.3)	1
Sleep disorders	2 (50.0)	14 (38.9)	1	6 (60.0)	3 (21.4)	0.092
Autonomic disturbance	2 (50.0)	20 (55.6)	1	3 (30.0)	7 (50.0)	0.421
Tumor presence	0	5 (13.9)	1	4 (40.0)	1 (7.1)	0.149
EEG (abnormal)	2 (50.0)	17 (47.2)	1	6 (60.0)	10 (71.4)	0.884
Brain MRI (abnormal)	2 (50.0)	18 (50.0)	1	5 (50.0)	5 (35.7)	0.78
CSF analysis (abnormal)	4 (100.0)	32 (88.9)	1	8 (80.0)	14 (100.0)	0.163
CSF cell count (abnormal)	2 (50.0)	28 (77.8)	0.543	6 (60.0)	8 (57.1)	1
CSF protein level (abnormal)	2 (50.0)	13 (36.1)	1	2 (20.0)	5 (35.7)	0.704
Antibody titer						
+	1 (25.0)	16 (44.4)	0.713	1 (10.0)	3 (21.4)	0.598
++	2 (50.0)	15 (41.7)		7 (70.0)	7 (50.0)	
+++	1 (25.0)	5 (13.9)		2 (20.0)	4 (28.6)	
Administration of HGG	3 (75.0)	34 (94.4)	0.277	6 (60.0)	9 (64.3)	1
Administration of corticosteroids	2 (50.0)	33 (91.7)	0.069	7 (70.0)	12 (85.7)	0.671
Administration of plasma exchange	0	4 (11.1)	1	0	1 (7.1)	1
Second-line immunotherapy	0	4 (11.1)	1	0	0	−
Without immunotherapy	0	0	−	1 (10.0)	0	0.417
Immunotherapy delay	1 (25.0)	13 (36.1)	1	4 (40.0)	5 (35.7)	1
Internal to immunotherapy	21 (17, 29)	18 (13, 35)	0.68	23 (10, 40)	23 (12, 30)	0.796
ICU admission	2 (50.0)	22 (61.1)	1	8 (80.0)	8 (57.1)	0.464
Mechanical ventilation	1 (25.0)	12 (33.3)	1	3 (30.0)	1 (7.1)	0.355
Long hospital stay	2 (50.0)	22 (61.1)	1	2 (20.0)	1 (7.1)	0.754
Relapse	1 (25.0)	9 (25.0)	1	1 (10.0)	7 (50.0)	0.107

IQR, interquartile range; NMDAR, N-methyl-D-aspartate receptor; GABABR, g-aminobutyric acid type B receptor; CSF, cerebrospinal fluid; EEG, electroencephalogram; MRI, magnetic resonance imaging; HGG, human gamma globulin; ICU, intensive care unit. +: antibody titer ≤1:10; ++: antibody titer >1:10 and ≤1:100; +++: antibody titer >1:100 as (+++).

## Discussion

In the present study, 15% of patients with anti-NMDAR, anti-LGI1, or anti-GABABR encephalitis died during a median follow-up of 18 months. The mortality rates of anti-NMDAR encephalitis, anti-LGI1 encephalitis, and anti-GABABR encephalitis were 10%, 2.8%, and 41.7%, respectively. The study results suggested that older age at onset, SE occurrence, AE antibody type, and ICU admission were associated with the risk of death. Additionally, age at onset and AE antibody type were identified as independent predictors of death in these patients.

Our study showed a general mortality rate of 15% in AE patients overall. In the subgroup with anti-NMDAR encephalitis, 10% of the patients died, which was in agreement with the 11.45% reported by Zhou et al. in West China (11). However, the mortality rate in our study was higher than the 5% reported in a cohort of patients with anti-NMDAR encephalitis in a multi-institutional observational study (7). This may be due to a treatment gap between developing countries and developed countries. Additionally, we found that the mortality rate was much higher in patients with anti-GABABR encephalitis than in those with the other two antibody types. One explanation may be that there is a higher prevalence of underlying cancer in the anti-GABABR subgroup (13, 18). It has been reported that the presence of cancer is associated with an increased risk of death in AE patients (10). In the present study, the mortality rate of anti-GABABR encephalitis (41.7%) was similar to the rates reported by Maureille et al. in France (41.0%) and by Lancaster et al. in the USA (40.0%) (19, 20). However, van Sonderen et al. reported a 2-year case fatality rate of 19% in patients with anti-LGI1 encephalitis, which was higher than the rate in our cohort (21). The mortality rate of anti-LGI1 encephalitis may have been underestimated due to the relatively short follow-up period compared with the study by Titulaer et al. (21).

We found that older age at onset increased the risk of death in patients with anti-NMDAR, anti-LGI1, and anti-GABABR encephalitis. Our finding was supported by Hong and her colleagues (10). In their study, all patients who died were over 45 years old, and they proposed that patients aged 45 years or older had an increased risk of death (10). This may be because older AE patients were more likely to have underlying tumors and develop systemic complications that could lead to death (13). According to the literature, admission to the ICU was also more common in AE patients aged ≥18 years than in those aged <18 years, indicating the relationship between age at onset and the severity of illness (22).

This study showed that AE patients in the death group were more likely to experience SE than those in the survival group. A recent study from West China also reported that refractory SE was one of the main causes of death in patients with anti-NMDAR encephalitis (11). The occurrence of SE has been reported as one of the most common reasons for ICU admission (13, 23). A history of SE also predicts poor outcomes and death in patients with all-cause encephalitis (24). However, de Montmollin et al. noted that the occurrence of SE during the ICU stay was not a prognostic factor for poor neurological outcome in patients with anti-NMDAR encephalitis (25). In the present study, admission to the ICU was identified as another important predictor of death. Prior research also supported our finding (11). ICU management in AE patients frequently involves long-term care. A longer ICU stay increases the risk of complications such as pneumonia, deep venous thrombosis, and multiple organ dysfunction syndrome (MODS), which may threaten the patient’s life (13, 18). A higher number of complications have been reported to be associated with a higher likelihood of death (10). Similarly, prior literature suggests that the lack of ICU admission was a prognostic factor for good outcomes in patients with anti-NMDAR encephalitis (7, 9). However, a cohort from West China did not find a relationship between admission to the ICU and long-term functional outcomes in anti-NMDAR encephalitis (22). Thus, well-designed studies with large sample sizes are required to confirm our findings.

Our data showed that AE patients in the death group were more likely to have an underlying tumor than those in the survival group. However, this difference was not statistically significant (*p* = 0.058), which may be explained partly by the relatively small sample size. Another possible explanation could be that approximately 50% of patients with anti-GABABR encephalitis were reported previously to have an underlying tumor; however, only 11% of patients in our cohort had tumors (19). As for anti-NMDAR encephalitis, a similarly reduced tumor rate has also been observed in a Chinese cohort (26, 27). It is well known that patients can benefit greatly from awareness and knowledge of AE (26, 28). However, there exists a knowledge gap regarding AE between China and developed countries. Thus, the differences may be a result of inadequate regular tumor screening and relatively short follow-up for some patients due to limited knowledge. We also observed a relatively low percentage of female patients in the anti-NMDAR encephalitis group, which may be explained partly by the reduced prevalence of ovarian teratoma in our cohort compared with Western cohorts. Tumor progression could contribute to an increased risk of death in anti-GABABR encephalitis (19, 29). Interestingly, the literature suggests the exciting possibility that the presence of tumors does not lead to a poor clinical outcome in patients with anti-NMDAR encephalitis (9, 22).

The present study has several limitations. First, the sample size in this cohort was relatively small, which may have led to limited statistical power. Relatedly, this small sample was drawn from a local tertiary hospital in Northeast China, and a regional selection bias may therefore exist. Second, the follow-up period was relatively short in some patients, which may cause the mortality rate to be underestimated in AE patients. These patients are still at risk of death over a longer period of time. Third, some patients with other types of antibodies, such as anti-Caspr2 antibodies, were excluded, which may limit the interpretation of the analysis results for the types of AE. Fourth, we did not assess the effect of tumor removal on the risk of death because data on the management of tumors were not available. Finally, the statistical significance threshold was not corrected for multiple comparisons, and variable selection for multivariable modeling according to univariate significance can be misleading (30, 31).

In summary, our data showed an overall mortality rate of 15% in patients with anti-NMDAR, anti-LGI1, or anti-GABABR encephalitis. Age at onset and type of AE antibody were identified as independent predictors of death in these patients.

## Data Availability Statement

The raw data supporting the conclusions of this article will be made available by the authors, without undue reservation.

## Ethics Statement

The studies involving human participants were reviewed and approved by the medical ethical committees of the First Hospital of Jilin University. Written informed consent from the participants’ legal guardian/next of kin was not required to participate in this study in accordance with the national legislation and the institutional requirements.

## Author Contributions

WL and RZ conceived and designed the study. RZ, HZ and XZ were involved in data acquisition. RZ and QC analyzed the data and wrote the manuscript. All authors contributed to the article and approved the submitted version.

## Conflict of Interest

The authors declare that the research was conducted in the absence of any commercial or financial relationships that could be construed as a potential conflict of interest.

## Publisher’s Note

All claims expressed in this article are solely those of the authors and do not necessarily represent those of their affiliated organizations, or those of the publisher, the editors and the reviewers. Any product that may be evaluated in this article, or claim that may be made by its manufacturer, is not guaranteed or endorsed by the publisher.
